# Jejunal and pancreatic transcriptomic adaptations underpin enhanced performance in broilers fed sugarcane bagasse-supplemented diets

**DOI:** 10.1186/s12864-026-12978-3

**Published:** 2026-05-22

**Authors:** Collins Amponsah Asiamah, Sarbast K. Kheravii, Sosthene Musigwa, Shu-Biao Wu, Sara de las Heras-Saldana

**Affiliations:** 1 School of Environmental and Rural Science, University of New England, Armidale, NSW 2351 Australia; 2 Animal Genetics and Breeding Unit, NSW Department of Primary Industries and Regional Development and University of New England, Armidale, NSW 2351 Australia

**Keywords:** Sugarcane bagasse, Jejunum, Pancreas, Transcriptome, Differentially expressed genes

## Abstract

**Background:**

This study investigated the transcriptomic changes in the jejunum and pancreas of broiler chickens fed a diet supplemented with sugarcane bagasse (SB) to elucidate the physiological response of broilers to insoluble dietary fiber.

**Results:**

A total of 168 0-day-old Ross 308 male broiler chicks were randomly allocated to two dietary treatments: a control diet or a diet supplemented with 2% SB, each consisting of six replicate pens (14 broilers/pen). Broilers were fed starter (d 0–10) and grower (d 11–24) diets, with SB added over the top to dilute the feed. Performance parameters were measured at d 10 and 24, and RNA sequencing was conducted on six broilers/treatment from jejunal and pancreatic tissues. Differentially expressed genes (DEGs) were identified (significant threshold at absolute log_2_ (fold change) ≥ 1 and *P* < 0.05) and functionally annotated using gene ontology (GO; gene counts ≥ 2 and *P* < 0.05 as significant) analysis. Compared with the control broilers, SB-fed broilers presented significantly (*P* < 0.05) greater weight gain and improved feed conversion ratio at the end of the grower phase. A total of 41 (18 upregulated and 23 downregulated) jejunal and 125 (36 upregulated and 89 downregulated) pancreatic DEGs were identified from the differential expression analysis. The SB-fed broilers showed coordinated upregulation of DEGs involved in nutrient transport (*TRPM3*, *SLC16A4*, *FFAR4*, and *RBP4A*), epithelial integrity (*WNT9A*, *GAL3ST2*, *TFF3*, and *AGR2*), immune activation (*DUOX2* and *MHCY6*), and growth regulation (*POU1F1*). Gene ontology enrichment further revealed significant activation of biological processes, including maintenance of the gastrointestinal epithelium, gastric acid secretion, sodium ion transport, and response to oxidative stress, in the pancreatic tissue.

**Conclusion:**

These findings reveal that SB supplementation triggers beneficial, tissue-specific transcriptomic adaptations that support nutrient uptake, epithelial repair, immune response, and oxidative balance, thereby enhancing growth. This study offers new insights into how broilers respond to dietary fiber supplementation at the transcriptomic level, supporting the strategic use of SB in sustainable poultry production.

**Supplementary Information:**

The online version contains supplementary material available at 10.1186/s12864-026-12978-3.

## Background

The poultry industry has made significant progress toward sustainability through various dietary and nutritional strategies. These include protein and energy modifications, increased grain particle size, dietary fiber inclusion, and the addition of feed additives [1–3]. These strategies enhance digestive efficiency, optimize nutrient utilization, and support gut health, thereby improving performance while minimizing environmental impact.

Poultry diets are formulated to balance protein and dietary energy by combining various ingredients, including legumes and cereal grains. However, the high demand for these ingredients for food and biofuel has increased their costs [4]. To alleviate this problem, alternative agricultural products, including wheat middlings, soy hulls, oil cakes, distillers dried grains with solubles, and sugar beet pulp, have been used in poultry diets [5–7]. These ingredients, however, contain high levels of dietary fiber, mainly composed of non-starch polysaccharides (NSP), lignin, and other indigestible carbohydrates [8]. Earlier studies considered dietary fiber an antinutritional factor due to its adverse effects on nutrient digestibility and performance in broiler chickens [9]. These adverse effects could result from the high amounts (187 and 375 g/kg) and the fiber sources used (pea fiber, wheat bran, or oat bran). However, recent research has shown that moderate inclusion of dietary fiber at 2 and 3% may positively affect the gastrointestinal tract (GIT) development, gizzard development, enzyme production, digestive physiology (nutrient digestion, fermentation, and absorption processes), intestinal integrity, beneficial microbiota, and immune functions [2, 6, 10]. These mechanisms collectively enhance nutrient utilization and growth performance of poultry [2, 6, 10].

Sugarcane bagasse (SB) is a highly fibrous byproduct of the sugar industry that has attracted interest as a potential dietary fiber source in poultry nutrition. It is composed of approximately 35% to 50% cellulose, 20% to 25% hemicellulose, and 15% to 25% lignin [11, 12]. It has been shown that SB may influence gut health and digestion through microbial fermentation and the production of short-chain fatty acids (SCFA), which serve as energy sources for intestinal epithelial cells in broiler chickens [13, 14]. Studies incorporating 2% SB into a coarsely ground corn-based diet revealed improved weight gain (WG) and feed conversion ratio (FCR), intestinal protein and starch digestibility, and upregulated digestive enzyme genes (*PGA5* and *PGC*) in the proventriculus at d 24 in broiler chickens [2, 15, 16]. Supplementing 2% SB in broiler diets increased the expression of digestive enzymes (*AMY2A* and *CELA1* ) in the pancreas, but *CAT1* and *GLUT2*, both nutrient transporters, were increased and decreased, respectively, in the jejunum [15]. Despite these highlighted benefits of dietary fiber, the molecular mechanisms by which SB supplementation, particularly in coarsely ground corn-based diet, affects nutrient absorption, intestinal integrity, immunity, and metabolic regulation remain poorly understood.

The jejunum is the primary site for nutrient absorption, transporter activity, intestinal barrier maintenance, and immune defense [17, 18], whereas the pancreas plays a critical role in the secretion of digestive enzymes to hydrolyze proteins, fats, and carbohydrates into monomers for absorption [19]. Exploring transcriptomic changes in these tissues may provide insight into the mechanisms underlying fiber-driven metabolic adaptations in broilers. Thus, this study employs RNA sequencing (RNA-seq) to investigate the transcriptomic profiles in the jejunum and pancreas of broilers fed diets supplemented with SB. This study hypothesizes that fiber supplementation would induce beneficial transcriptomic changes that enhance digestive function by modulating differential gene expression related to nutrient transport, intestinal integrity, enzyme activity, immunity, and metabolism in broiler chickens. This research offers novel insights into the molecular mechanisms underlying fiber utilization in broilers, thereby contributing to the development of improved dietary strategies for sustainable poultry production.

## Materials and methods

### Experimental design, broiler management, and diet

The experimental design, broiler management, and diet preparation followed the procedures described by Kheravii et al. [15], with slight modifications in this study. Briefly, 168 0-day-old Ross 308 male broiler chicks (Baiada Hatchery, Tamworth, New South Wales, Australia) were weighed and randomly assigned to 12 floor pens, each measuring 75 cm × 120 cm, with 14 broilers per pen (stocking density approximately 15.56 broilers/m²). Male chicks were used to minimize sex-related biological variations and to improve comparability among dietary treatments. The broilers were randomly allocated to two dietary treatments: 0% or 2% sugarcane bagasse (SB) in a coarsely ground corn (3,576 μm) diet, with six replicates per treatment. The 2% SB inclusion level was selected based on previous broiler studies reporting benefits to performance and digestive function at this practical low inclusion rate [2, 15, 16]. Diets were formulated based on Ross 308 specifications [20]. The starter (d 0 to 10) and grower (d 11 to 24) diets were prepared, with SB included by diluting the complete feed with 2% SB added on top. All diets were thoroughly mixed and cold-pelleted at 65 °C. Each pen was equipped with a single tube feeder and 2 nipple drinkers. Broilers had *ad libitum* access to feed and water. Housing conditions, including lighting, temperature, and relative humidity, adhered to Ross 308 management guidelines [20]. The room temperature was kept around 30 °C for the first 3 days and then gradually lowered by 2–3 °C each week until reaching 21 °C by d 24. During the first week, a 23-hour light and 1-hour dark cycle was used, followed by a 20-hour light and 4-hour dark cycle afterward. Relative humidity was maintained between 50% and 60% throughout the entire experiment. The SB used was supplied by the FCR Consulting Group, Brisbane. Its chemical composition was analyzed on an “as is” basis for total NSP and lignin, following the methods described by Englyst et al. [21] and Kirk and Obst [22], respectively. The SB contained 6.1 g/kg free sugar, 191 g/kg lignin, 534 g/kg insoluble NSP, and 1.9 g/kg soluble NSP [15]. The ingredients and calculated nutrient compositions of the experimental diets are detailed in Table 1 [15].

**Table 1:** Composition and calculated nutrient content of base diet (%)

Ingredients	Starter (d 0–10)		Grower (d 11–24)	
	Control	SB	Control	SB
Corn	60.60	59.39	62.30	61.05
Soybean meal	32.60	31.95	29.30	28.71
Meat and bone meal	3.00	2.94	3.60	3.53
Canola oil	0.64	0.63	1.91	1.87
Sugarcane bagasse	0.00	2.00	0.00	2.00
Limestone	0.97	0.95	0.81	0.79
Dicalcium phosphate	0.61	0.60	0.27	0.26
Phytase^a^	0.01	0.01	0.01	0.01
Salt	0.15	0.15	0.16	0.16
Na bicarbonate	0.22	0.22	0.20	0.20
Vitamin-mineral^b^	0.20	0.20	0.20	0.20
Choline	0.11	0.11	0.10	0.10
L-lysine HCl 784	0.31	0.30	0.23	0.23
D, L-methionine	0.39	0.38	0.34	0.33
L-threonine	0.20	0.20	0.15	0.15
TiO_2_	0.00	0.00	0.50	0.49
Total	100.00	100.00	100.00	100.00
Nutrients				
ME (kcal/kg)	3,000	2,940	3,100	3,038
Crude protein	22.20	21.76	21.00	20.58
Crude fat	2.85	2.79	4.14	4.06
Crude fiber	2.07	2.95	2.01	2.89
SID arginine	1.37	1.34	1.27	1.24
SID lysine	1.28	1.25	1.15	1.13
SID methionine	0.68	0.67	0.62	0.61
SID methionine + Cysteine	0.95	0.93	0.87	0.85
SID tryptophan	0.24	0.24	0.23	0.23
SID isoleucine	0.86	0.84	0.81	0.79
SID threonine	0.86	0.84	0.77	0.75
SID valine	0.99	0.97	0.94	0.92
Starch	35.80	35.08	36.80	36.06
NSP soluble	0.43	0.43	0.40	0.40
NSP insoluble	5.64	6.60	5.45	6.41
Calcium	0.96	0.94	0.87	0.85
Available phosphorus	0.48	0.47	0.44	0.43
Sodium	0.16	0.16	0.16	0.16
Chloride	0.25	0.25	0.24	0.24
Choline	0.17	0.17	0.16	0.16

*SID* Standard ileal digestible, *NSP* Non-starch polysaccharide, *ME* Metabolizable energy, *SB* Sugarcane bagasse

^a﻿^Phyzyme XP5000G (100 g/mt) Dupont

^b﻿^Vitamin-mineral concentrate supplied per kilogram of diet: retinol, 12,000 IU; cholecalciferol, 5,000 IU; tocopheryl acetate, 75 mg, menadione, 3 mg; thiamine, 3 mg; riboflavin, 8 mg; niacin, 55 mg; pantothenate, 13 mg; pyridoxine, 5 mg; folate, 2 mg; cyanocobalamine, 16 µg; biotin, 200 µg; cereal-based carrier, 149 mg; mineral oil, 2.5 mg; Cu (sulphate), 16 mg; Fe (sulphate), 40 mg; I (iodide), 1.25 mg; Se (selenate), 0.3 mg; Mn (sulphate and oxide), 120 mg; Zn (sulphate and oxide), 100 mg; cereal-based carrier, 128 mg; mineral oil, 3.75 mg

### Performance and sample collection

Broilers and leftover feed in each pen were weighed at the end of the starter (d 10) and grower (d 24) phases. On d 24, one broiler from each pen was randomly selected and euthanized by cervical dislocation for proximal jejunum and pancreatic tissue collection. An approximately 2 cm segment from each tissue was excised, rinsed with sterile 4 °C phosphate-buffered saline (PBS), and placed into a 2 mL Eppendorf tube. The PBS was kept at 4 °C during sampling by refilling a small working bottle from a refrigerated stock as needed. The samples were snap-frozen in liquid nitrogen and stored at -80 °C for RNA extraction.

### RNA extraction and sequencing

Total RNA was extracted from each sample using TRIsure™ (Cat# BIO-38032, Bioline, Sydney, Australia) following the manufacturer’s instructions. The RNA concentration and purity were measured using a NanoDrop ND-8000 spectrophotometer (Thermo Fisher Scientific, Waltham, MA), and RNA integrity was assessed with an Agilent 2100 Bioanalyzer and the RNA 6000 Nano Kit (Agilent Technologies, Inc., Waldron, Germany). The RNA samples with A260/230 values > 1.8, A260/280 values ranging from 1.8 to 2.0, and RNA integrity numbers (RIN) > 7.5 were considered high quality. In the present study, the RIN of the samples ranged from 7.5 to 10. The RNA samples were processed and sequenced by the Australian Genomic Research Facilities (Melbourne, Vic., Australia). Image analysis was performed in real-time by the NovaSeq Control Software v1.7.5 and Real-Time Analysis v3.4.4, which were run on the instrument computer for real-time base calling. The Illumina DRAGEN BCL Convert 07.021.624.3.10.8 pipeline was subsequently used to generate the FASTQ sequencing files.

### Transcriptome mapping and assembly

The quality of the raw sequencing reads was evaluated via FastQC v.0.12.0 (https://www.bioinformatics.babraham.ac.uk/projects/fastqc/) [23], and low-quality reads, and adapters were filtered via Trimmomatic v.0.39 software [24]. Reads containing adapter sequences, more than 10% unknown nucleotides, and low Phred scores (Q < 30) were removed to ensure high-quality sequence data. Cleaned reads were then aligned to the *Gallus gallus* reference genome (GRCg7b) using STAR v.2.7.9 software [25]. The mapped reads were assembled using HTSeq v.2.0.3 [26] to generate a gene count matrix for downstream analyses.

### Differential expression analysis and functional annotation

Before performing the differential expression analysis, genes with low-expression levels were filtered, and gene expression values were normalized using the filterByExpr and calcNormFactors functions based on the trimmed mean of M-values method in the edgeR package v.4.0.16 [27]. A principal component analysis (PCA) was conducted to explore variation across samples and detect potential outliers that could influence downstream analysis. Outliers, identified as samples deviating significantly from their respective diet groups, confirmed by lower performance in the pancreas and higher RNA concentration in the jejunum, were excluded from further analyses. A generalized linear model (GLM) was fitted to the data using the glmQLFit function in edgeR v.4.0.16 [28] to assess differential gene expression between the control and SB diets. Differentially expressed genes (DEGs) were identified using an absolute log_2_(fold change) ≥ 1 and *P* < 0.05 as significance thresholds.

Pathway and functional enrichment analyses were conducted using the Database for Annotation, Visualization, and Integrated Discovery (DAVID v.6.8) [29] to explore the gene ontology (GO) and pathways, gaining insight into the biological significance of the DEGs. The GO analysis categorizes DEGs into biological processes, cellular components, and molecular functions. Kyoto Encyclopedia of Genes and Genomes (KEGG) pathway enrichment identified significantly affected metabolic and regulatory pathways. Both analyses were performed, with a threshold of *P* < 0.05 and gene counts ≥ 2. All functional annotations were based on *G. gallus* (GRCg7b) reference genome annotations.

### Statistical analysis

All data analyses were conducted in R software (v.4.1.2, R Core Team 2021, R Foundation for Statistical Computing, Vienna, Austria) [30]. The average WG, feed intake (FI), and FCR were estimated for each stage. Performance variables (WG, FI, and FCR) were tested for normality using the Shapiro-Wilk test and for homogeneity of variances using Levene’s test. For each response, a linear model was fit with diet as the factor:

$${Y}_{i}={\beta}_{0}+{\beta}_{1}\cdot\:die{t}_{i}+{\epsilon}_{i}$$

where *Y*_*i*_ is the response variable (WG, FI, and FCR); *β*_*0*_ is the intercept; *β*_*1*_ is the coefficient for *i* diet (control and SB); *𝜀*_*i*_ is the residual error term, assumed *ε*_*i*_ ~ N(0,σ^2^). The estimated marginal means (emmeans) and their standard errors were obtained via the emmeans R package (v.1.10.4). Diet contrasts (control vs. SB) were tested using pairs (emmeans(model, ~ diet). Significance was set at a threshold of *P* < 0.05.

## Results

### Broiler performance

The effects of SB on broiler performance are shown in Table 2. The inclusion of 2% SB had no significant effect on starter performance. By d 24, however, SB supplementation significantly (*P* < 0.05) increased WG and improved FCR compared with those of the control broilers.

**Table 2:** Effects of SB on broiler performance

Diet	D 0–10			D 11–24			D 0–24		
	WG (g/bird)	FI (g/bird)	FCR	WG (g/bird)	FI (g/bird)	FCR	WG (g/bird)	FI (g/bird)	FCR
Control	276.7	290.3	1.05	1,158	1,542	1.33	1,434	1,800	1.26
SB	267.4	286.6	1.07	1,233	1,579	1.28	1,501	1,837	1.22
Pooled SEM	3.925	4.360	0.009	10.754	21.050	0.011	12.830	22.340	0.009
*P-*value	0.126	0.569	0.089	0.001	0.240	0.010	0.004	0.270	0.043

*SB* Sugarcane bagasse, *SEM* Standard error of mean, *WG* Weight gain, *FI* Feed intake, *FCR* Feed conversion ratio, *D*  Day

### RNA-seq data analysis

Sequencing and mapping quality metrics for each tissue and diet are summarized in Table 3. On average, each group yielded over 7 million clean reads, with Q30 scores exceeding 91% and GC content from 44% to 55%. Mapping rates were high, with total and unique alignment rates above 84% and 71%, respectively.

**Table 3:** Sequencing output and mapping statistics for jejunum and pancreas RNA-seq libraries

Tissue	Diet^a^	Mean raw reads	Mean clean reads	Total mapped rate (%)	Unique mapped rate (%)
Jejunum	Control	9,021,123	7,003,375	89.9	86.9
	SB	15,789,928	12,222,939	89.2	86.2
Pancreas	Control	12,826,290	9,969,542	84.1	71.5
	SB	27,632,148	21,496,647	84.8	73.7

^a^SB = sugarcane bagasse

### Identification of differentially expressed genes

**Fig. 1:**
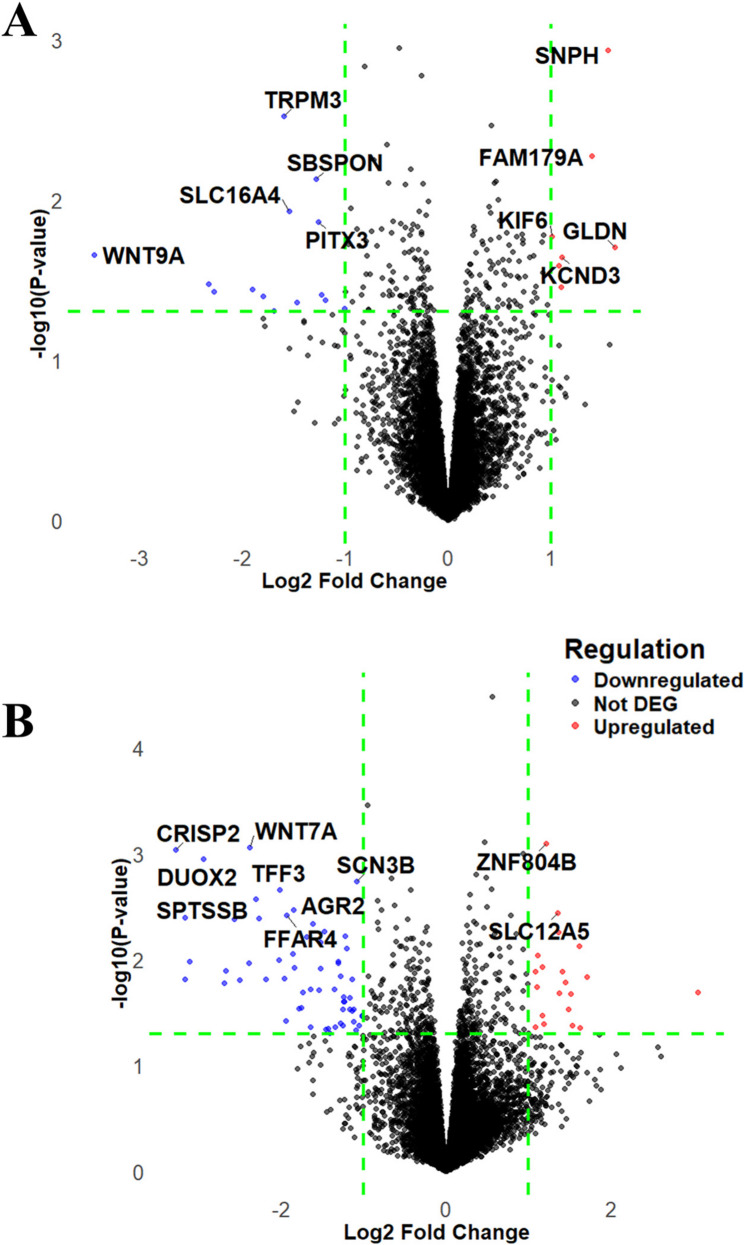
Volcano plots of DEGs in control vs. SB in jejunum (**A**) and pancreas (**B**) of broiler chickens. The X-axis shows the log_2_ fold change of DEGs between the two groups. The Y-axis indicates the -log_10_(*P*-value) of gene expression variation. Each dot in the volcano plot represents a gene; red dots represent upregulated DEGs, blue dots represent downregulated DEGs in the control diet condition, and black dots represent the non-significant genes

Comparative analysis between the control vs. SB contrast revealed significant DEGs in the jejunum and pancreas, with key results visualized in Fig. 1. In the jejunum, 41 DEGs were identified, of which 18 were upregulated and 23 were downregulated (Supplementary Table 1, Fig. 1A). Among the 125 DEGs identified in the pancreas, 36 were upregulated and 89 were downregulated (Supplementary Table 2, Fig. 1B). The top 15 annotated upregulated and downregulated DEGs in each tissue, with their functions, are shown in Tables 4 and 5.

**Table 4:** Functions of the top 15 upregulated and downregulated DEGs in the jejunum of broilers fed the control diet compared to those fed the SB diet

Gene symbol	logFC^a^	*P*-value	Direction^b^	Functions	References
*SNPH*	1.56	0.001	Upregulated	Essential for mitochondrial stabilization in mice	[31, 32]
*FAM179A*	1.40	0.005	Upregulated	Fuses anaplastic lymphoma kinase gene in plasma cell-free DNA in humans	[33]
*KIF6*	1.01	0.017	Upregulated	Transports vesicles, organelles, protein complexes, and messenger ribonucleic acids toward the cell nucleus in humans	[34]
*GLDN*	1.62	0.020	Upregulated	Plays vital roles in cellulose degradation, cell motility, and acquisition of trace amounts of Ca^2+^ and Mg^2+^ in *Cytophaga hutchinsonii* bacterium	[35]
*KCND3*	1.11	0.023	Upregulated	Related to the growth and development of Wenchang chickens.	[36]
*RASSF10*	1.08	0.026	Upregulated	Contributes to tumorigenesis by promoting epithelial-mesenchymal transition induced by *TGFβ* in humans	[37]
*TRPM3*	-1.59	0.003	Downregulated	Triggers Ca²⁺ influx, promotes insulin secretion, and maintains glucose homeostasis	[38, 39]
*SBSPON*	-1.28	0.007	Downregulated	Plays a role in wound healing in planarian	[40]
*SLC16A4*	-1.55	0.012	Downregulated	Transports short-chain monocarboxylate, hormones, nutrients, and amino acids across the plasma membrane	[41]
*PITX3*	-1.26	0.014	Downregulated	Regulates midbrain dopaminergic neuron terminal differentiation in humans	[42]
*WNT9A*	-3.44	0.022	Downregulated	Promotes epithelial regeneration and barrier integrity in mice	[43]
*PRDM8*	-2.33	0.033	Downregulated	Regulates the motor neuron-oligodendrocyte precursor cells switch and allocation in Zebrafish, involved in embryo development in chickens	[44, 45]
*VEPH1*	-1.90	0.036	Downregulated	Mediates tumor suppressing activity through regulation of cell proliferation, migration, and invasion in human	[46]
*ZNFY1*	-2.28	0.038	Downregulated	Binds DNA to regulate gene expression	[47]
*MHCY6*	-1.77	0.039	Downregulated	Crucial for adaptive immunity, present processed antigens to activate T cells to clear avian influenza virus in ducks	[48]

^a^logFC = log_2_ fold change

^b﻿^Direction: Indicates whether the gene is upregulated or downregulated. Upregulated genes are highly expressed in broilers fed the control diet than in SB-fed broilers, whereas downregulated genes are expressed at lower levels in control-fed broilers compared to those fed the SB diet

**Table 5:** Functions of the top 15 upregulated and downregulated DEGs in the pancreas of broilers fed the control diet compared to those fed the SB diet

Gene symbol	logFC^a^	*P*-value	Direction^b^	Functions	References
*ZNF804B*	1.21	0.001	Upregulated	Related to egg laying in chickens	[49]
*SLC12A5*	1.35	0.004	Upregulated	Involved in active transport, passive transport, metal ion transport, and ion exchange.	[50]
*CFAP45*	1.36	0.005	Upregulated	Supports mammalian ciliary and flagellar beating via an adenine nucleotide homeostasis module.	[51]
*RFLNA*	1.61	0.007	Upregulated	Binds and regulates filamin proteins to the actin cytoskeleton, regulating cell shape and motility	[52]
*DUSP6*	1.11	0.009	Upregulated	Mediates T cell receptor-engaged glycolysis in mice	[53]
*CGTL*	1.33	0.011	Upregulated	Exports glutamate, and imports cysteine into cells of chickens	[54]
*PLEKHM3*	1.16	0.012	Upregulated	Involved in muscle differentiation and development in chickens and geese	[55, 56]
*WNT7A*	-2.38	0.001	Downregulated	Accelerates and augments skeletal muscle regeneration and to ameliorate dystrophic progression	[57]
*CRISP2*	-3.28	0.001	Downregulated	Crucial for male fertility and sperm function in animals.	[58]
*DUOX2*	-2.94	0.001	Downregulated	Act as a first line of the defense system in *Drosophila*	[59]
*SCN3B*	-1.08	0.002	Downregulated	Regulates electrically excitable cells, feather formation, and development in chickens	[60, 61]
*TFF3*	-2.01	0.002	Downregulated	Safeguards, restores, and ensures the continuity of the GIT epithelium	[62].
*SPTSSB*	-2.30	0.003	Downregulated	Regulates serine palmitoyltransferase complex affinity	[63]
*AGR2*	-1.85	0.003	Downregulated	Preserves goblet cell health, function, and mucus barrier integrity to maintain gut homeostasis	[64, 65]
*FFAR4*	-1.93	0.004	Downregulated	Senses long-chain fatty acids, mediates anti‐inflammatory signaling and insulin secretion	[66, 67]

^a﻿^logFC = log_2_ fold change

^b﻿^Direction: Indicates whether the gene is upregulated or downregulated. Upregulated genes are highly expressed in broilers fed the control diet than in SB-fed broilers, while downregulated genes are expressed at lower levels in control-fed broilers compared to those fed the SB diet

Notably, several DEGs identified in both tissues are associated with key physiological processes relevant to nutrient metabolism and intestinal health. In the jejunum, upregulated DEGs, including *TRPM3*, *WNT9A*, and *GAL3ST2*, are involved in mineral transport, epithelial renewal, and mucin glycosylation, respectively. In the pancreas, upregulated DEGs including *DUOX2*, *TFF3*, and *POU1F1* are linked to redox signaling, mucosal protection, and growth regulation, respectively.

### Gene ontology and KEGG pathway

**Fig. 2:**
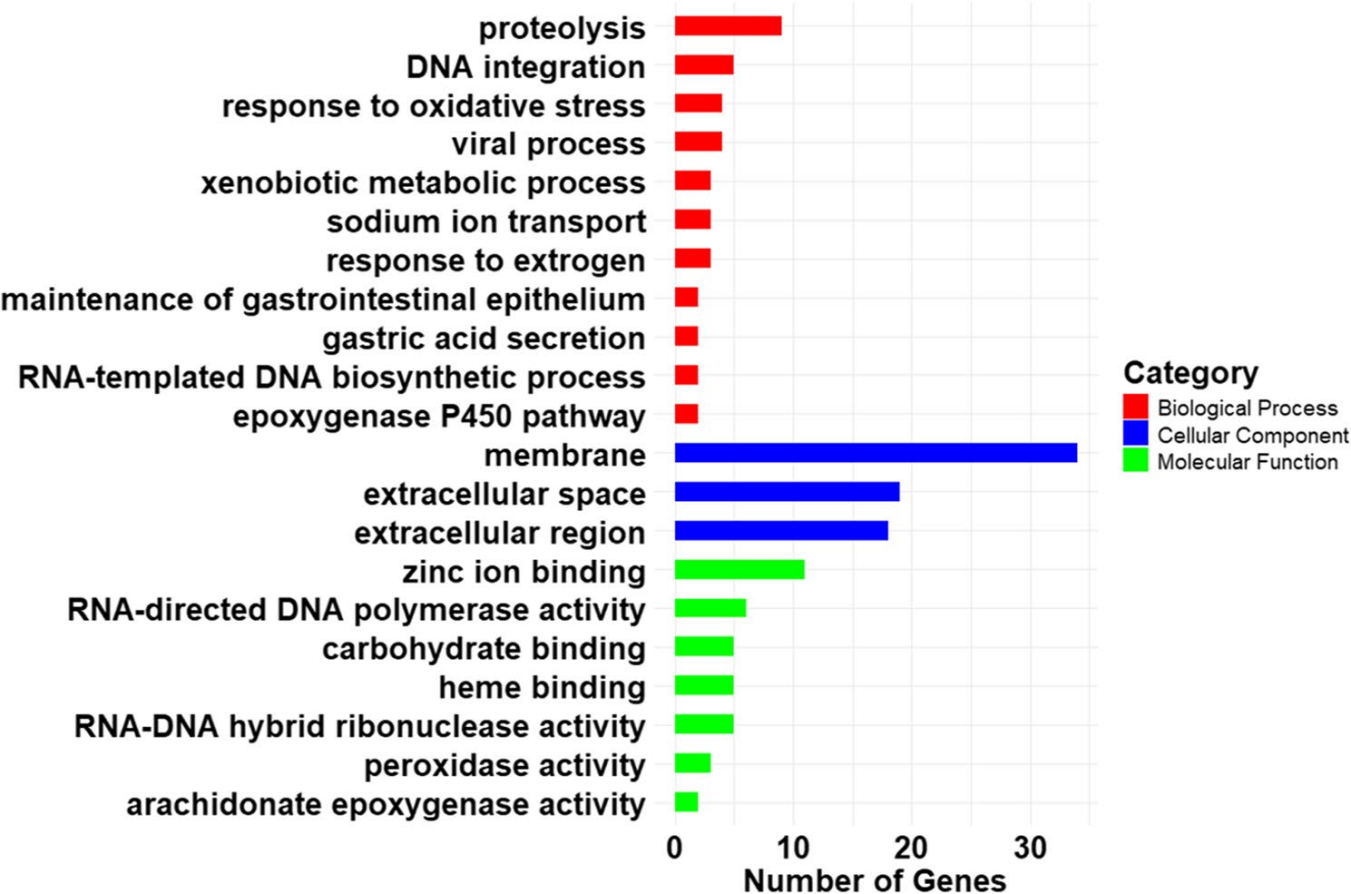
Gene ontology enrichment analysis of the DEGs in the pancreas. Enriched GO terms are on the y-axis, and the number of genes is on the x-axis

A functional enrichment analysis was conducted to investigate the functions of the DEGs. Using the applied thresholds, four GO terms were significantly (*P* < 0.05) enriched in the jejunum (Table 6). In the pancreas, eleven terms were significantly enriched in biological process, three terms in cellular component, and seven terms in molecular function (Fig. 2, Supplementary Table 3). The significantly enriched GO terms were mainly associated with processes involved in epithelial maintenance, ion transport, redox regulation, and metabolic activity. In the pancreas, enrichment of maintenance of gastrointestinal epithelium, sodium ion transport, and response to oxidative stress suggests transcriptional changes for improved digestion, epithelial protection, and redox balance under SB supplementation.

**Table 6:** Gene ontology enrichment analysis of the DEGs in the jejunum

Category	Term	Count	*P*-value	Genes
Biological Process	GO:0061045 ~ negative regulation of wound healing	2	0.022	*AJAP1*, *LOC121109863*
	GO:0051262 ~ protein tetramerization	2	0.037	*TRPM3*, *KCND3*
	GO:0045109 ~ intermediate filament organization	2	0.058	*LOC121109863*, *KRT24*
Cellular Component	GO:0016020 ~ membrane	15	0.002	*AJAP1*, *GAL3ST2*, *CCR8L*, *TRPM3*, *KCND3*, *SLC16A4*, *SNPH*, *LOC121106936*, *LOC112530310*, *LOC121106487*, *LOC112531163*, *LOC121109863*, *LOC107050135*, *ENSGALG00010005303*, *ENSGALG00010012331*
	GO:0005881 ~ cytoplasmic microtubule	2	0.065	*FAM179A*, *SNPH*
Molecular function	GO:0008017 ~ microtubule binding	3	0.052	*FAM179A*, *LOC121109863*, *KIF6*

However, the present study did not detect any biological pathways that significantly responded to the treatments.

## Discussion

This study investigated the impact of SB on broiler performance and transcriptome profiles in the jejunal and pancreatic tissues of both control and SB-supplemented broilers, aiming to understand the molecular basis of their performance. The study hypothesized that fiber supplementation would trigger beneficial transcriptomic changes that enhance digestive function by modulating DEGs related to nutrient transport, intestinal integrity, enzyme activity, immunity, and metabolism in broiler chickens. The findings reveal the upregulation of DEGs involved in mineral and fermentative energy uptake, epithelial maintenance, growth signaling, and immune regulation in broilers on the SB diet, which may contribute to improved performance. Therefore, the hypothesis that fiber supplementation modulates DEGs involved in intestinal integrity, nutrient transport, and other biological processes that may underlie the observed improved performance was accepted.

In this study, the addition of 2% SB to a corn-based diet did not affect starter-phase performance (d 0–10), but by d 24, it significantly increased WG and improved FCR. Because SB was added on top of the complete diet, calculated nutrient density was slightly diluted; however, performance improvements (higher WG and improved FCR) occurred without a significant increase in FI, suggesting a net positive functional effect of SB rather than an effect driven by minor nutrient differences. The lack of early effects likely reflects the broilers’ gradual physiological adaptation to the increased dietary fiber load. By the end of the grower period, SB‐fed broilers gained 75 g more weight and exhibited 5.2 points (3.9%) lower FCR compared to the control. These improvements align with earlier reports that the inclusion of moderate levels of insoluble fiber, such as SB, oat hulls, and soybean hulls, stimulates gizzard development and function and improves feed efficiency and nutrient digestibility, leading to better nutrient utilization and improved FCR and WG in broiler chickens [6, 16, 68]. The enhanced performance observed in SB-fed broilers is supported by the upregulation of several DEGs involved in physiological and biological processes that promote nutrient absorption, maintain epithelial integrity, and strengthen immune responses, as discussed below.

The present study explored transcriptomic changes in the jejunum and pancreas, given their central roles in digestion, absorption, and nutrient metabolism. The jejunum is the main site of nutrient absorption, transporter activity, and mucosal immune regulation [17, 18], while the pancreas secretes digestive enzymes that regulate nutrient metabolism [19]. Although SB is an insoluble fiber, its inclusion may influence the function of these organs through microbial fermentation processes in the hindgut. The fermentation of SB produces SCFAs such as acetate, propionate, and butyrate, which serve as an energy source for enterocytes, stimulate digestive enzyme activity, enhance nutrient absorption, and modulate immune responses [13, 14, 69]. These microbially derived metabolites may, therefore, contribute to the transcriptional changes observed in the jejunum and pancreas, which may support improved performance in SB-fed broilers.

The SB-supplemented diet induced expression of several nutrient transporters in the jejunum and pancreas, suggesting enhanced nutrient absorption. Transient receptor potential melastatin-3 (*TRPM3*) is a non-selective cation channel permeable to Ca²⁺, Na⁺, Mg²⁺, and Mn²⁺ [70, 71]. The *TRPM3* responds to pregnenolone sulfate by triggering intracellular Ca²⁺ influx, which supports insulin secretion and maintains glucose homeostasis in non-avian species [39, 72]. While SB provides essential minerals, such as Ca, Mg, K, and P [73], its fibrous components can bind minerals, potentially reducing their bioavailability [74]. Therefore, the observed upregulation of *TRPM3* in the jejunum of SB-fed broilers may indicate improved molecular regulation of intestinal mineral uptake and enterocyte Ca²⁺ signaling, to help maintain mineral balance. Similarly, solute carrier family 16 member 4 (*SLC16A4*), also known as monocarboxylate transporter 5 (*MCT5*, previously *MCT4*), which is essential for the transport of short-chain monocarboxylates (lactate, pyruvate, and ketone bodies), hormones, nutrients, and amino acids [41], was upregulated in SB-fed broilers. The high insoluble fiber content of SB increases hindgut microbial fermentation and elevates intestinal SCFA concentrations, promoting energy regulation, mucosal integrity, immune homeostasis, and immune maturation [13, 69]. Thus, the upregulation of *SLC16A4* in the jejunum of SB-fed broilers may enhance SCFA uptake into enterocytes, supporting epithelial energy metabolism. In the pancreas, SB supplementation upregulated free fatty acid receptor 4 (*FFAR4*, also known as *GPR120*), a G-protein-coupled receptor for long-chain fatty acids that enhances glucose-stimulated insulin secretion and mediates anti-inflammatory signaling in pancreatic islets [66]. The increased *FFAR4* expression in SB-supplemented broilers may reflect improved pancreatic sensing of fermentative lipids derived from the metabolism of insoluble fiber, supporting adaptive insulin secretion. This mechanism helps maintain glucose homeostasis, which may ensure an adequate energy supply and support improved growth in SB-fed broilers. Retinol-binding protein 4 A (*RBP4A*, also *RBP4*) is the principal plasma carrier of retinol (vitamin A), which is transported from hepatic and adipose tissues to peripheral tissues [75]. In this study, *RBP4A* was significantly upregulated in the pancreas of SB-supplemented broilers, indicating potential increased retinol transport and mobilization to support retinoid-dependent biological processes such as growth regulation, immune function, and epithelial integrity [75]. In chickens, *RBP4A* participated in lipid metabolic pathways in breast muscle [76]. Thus, upregulated *RBP4A* supports systemic vitamin A homeostasis and energy metabolism, which may contribute to enhanced performance in SB-supplemented broilers. These adaptations enable the recovery of more usable energy, vitamins, and minerals from the SB-diet for various biological processes, which may underlie the superior growth observed in these broilers.

Maintaining jejunal and pancreatic integrity under abrasive fiber loads requires rapid cell renewal and a robust mucus barrier [77, 78]. The gene WNT family member 9 A (*WNT9A*) promotes intestinal stem-cell proliferation and crypt-villus homeostasis, and is involved in the immune response during disease infection in chickens and mice [43, 79]. The high insoluble fiber content of SB may induce mild mechanical stimulation in the hindgut; thus, *WNT9A* upregulation in the jejunum of SB-fed broilers likely accelerates intestinal stem cell proliferation and mucosal regeneration, enhancing nutrient absorption. Concurrently, upregulation of galactose-3-O-sulfotransferase 2 (*GAL3ST2*), which catalyzes sulfation of mucin glycans [80], suggests increased regulation of mucin biosynthesis in the jejunum of SB-fed broilers. Sulfated glycans form a protective mucus barrier that shields the intestinal epithelium from abrasion and pathogen contact [81]. Also, somatomedin B and thrombospondin type 1 domain-containing (*SBSPON*), implicated in wound healing in planarians [40], was upregulated in the jejunum of SB-fed broilers. This increased expression may reflect activation of tissue repair and regenerative processes in response to stimulation from the insoluble-fiber diet. Such molecular responses may support epithelial maintenance and mucosal resilience under dietary SB supplementation, thereby supporting overall gut function during fiber digestion. A trefoil factor family member, *TFF3*, is a biologically active peptide that, together with mucins, contributes to epithelial protection under mechanical and inflammatory stress [62]. Its upregulation in the pancreas of SB-fed broilers may reflect a transcriptional response associated with epithelial protection or anti-inflammatory regulation. Additionally, an endoplasmic reticulum resident secretory protein, anterior gradient 2 (*AGR2*), was upregulated in the pancreas of SB-supplemented broilers. This protein is crucial for the health and function of goblet cells, as well as for maintaining mucus barrier integrity and gut homeostasis [64, 65]. It is possible that the increased *AGR2* may have contributed to the protection of the GIT mucosal barrier from inflammation while maintaining its integrity during mechanical stimulation from SB-high fiber. Collectively, these transcriptomic changes suggest that SB supplementation modulates genes involved in epithelial maintenance and mucus regulation, potentially contributing to improved gut function and overall broiler performance.

Sugarcane bagasse contains bioactive compounds, including xylooligosaccharides (from hemicellulose), which are prebiotics, as well as phytogens such as phenolics and flavonoids [82–84] with antimicrobial, anti-inflammatory, and antioxidant properties that enhance immune response and gut health in broilers [85, 86]. These compounds can modulate the gut environment and the expression of immune-related genes in broilers. The SB-fed broilers in this study exhibited upregulated expression of a major histocompatibility complex, *MHCY6*, in both the jejunum and pancreas, suggesting enhanced antigen exposure to T cells and adaptive immune response in chickens [48]. Dual oxidase 2 (*DUOX2*, a member of the NADPH oxidase family), which produces hydrogen peroxide (H_2_O_2_), a key mediator of redox signaling and mucosal innate immunity [87], was upregulated in the pancreas of SB-fed broilers. The *DUOX2* enzymes act as a first line of defense by producing microbicidal reactive oxygen species (ROS) and are activated by high Ca^2+^ concentrations in the cytoplasm [59]. Studies have confirmed that *DUOX2* is expressed throughout the digestive tract of mammals [88, 89]. It was reported that SB increases microbial fermentation in the hindgut and alters gut microbiota composition [13, 90], potentially stimulating immune pathways along the gut-pancreas axis. Therefore, the higher expression of *DUOX2* in the pancreas may reflect a beneficial adaptive response to increased microbial fermentation associated with fiber-rich diets. Moreover, increased Ca²⁺ influx due to upregulation of *TRPM3* in the jejunum may initiate *DUOX2*-mediated H_2_O_2_ production, contributing to redox-sensitive transcription and innate immune defense. Conversely, the expression of cystine/glutamate transporter-like (*CGTL*) and eosinophil peroxidase (*EPX*) genes involved in immune defense and redox balance was downregulated in the pancreas of SB-fed broilers. While *CGTL* mediates the exchange of intracellular glutamate for cystine, facilitating glutathione synthesis and epithelial redox homeostasis [54], *EPX* generates enzyme-based antimicrobial oxidants at inflammatory sites [91]. The reduced expression of these genes in SB-fed broilers may indicate a reduced reliance on innate antioxidant and enzyme-based defenses, possibly due to improved gut barrier integrity as discussed above. Together, these transcriptomic changes suggest that SB supplementation modulates redox and immune signaling, potentially improving mucosal defense and overall broiler performance. Moreover, the bioactive compounds in SB may reduce oxidative activities and enhance the immune response of broilers, as previously reported by Lumsangkul et al. [92] in *Nile tilapia* fed SB powder, which upregulated cytokines and antioxidants in the liver and intestine.

Adaptations in growth-regulatory signaling may also underlie the improved performance observed in SB-fed broilers. In this study, pituitary-specific positive transcription factor 1 (*POU1F1*), which controls growth hormone, prolactin, and thyroid-stimulating hormone β subunit expression [93, 94], was unexpectedly elevated in the jejunum of SB-fed broilers. In chickens, *POU1F1* expression has been positively associated with growth traits [95, 96]. Although its official role is in the pituitary, *POU1F1* upregulation in the jejunum of SB-fed broilers may reflect an adaptive entero-pituitary feedback loop aimed at sustaining growth hormone and prolactin-mediated growth processes. This mechanism could help maintain growth trajectories, suggesting SB’s potential to enhance nutrient and energy utilization, as shown by Kheravii et al. [16].

GO functional enrichment analysis revealed several key terms in the jejunum and pancreas. The enrichment of GO:0030277 ~ maintenance of gastrointestinal epithelium (enriched with *TFF3* and *RBP4A*) and GO:0001696 ~ gastric acid secretion (enriched with *GHRL* and *SLC9A4*) in the pancreas of SB-fed broilers highlights a coordinated physiological response. While the former maintains the integrity of the digestive tract’s epithelial barrier, the latter controls the release of hydrochloric acid to improve digestion. Furthermore, the enrichment of GO:0006814 ~ sodium ion transport in the pancreas of SB-fed broilers, driven by the upregulation of *SLC9A4*, *SLC9A3*, and *SCN3B*, likely enhanced sodium (Na^+^) homeostasis. The *TFF3* gene works with mucins to protect the GIT epithelium from mechanical and inflammatory damage [62]. The *RBP4A* gene enhances retinol uptake to support retinoid-dependent processes, such as growth regulation, immune function, and epithelial health [75]. The increased expression of these DEGs in SB-fed broilers indicates a coordinated molecular response to maintain epithelial integrity and function in response to dietary fiber. Ghrelin (*GHRL*), a peptide hormone primarily produced in the stomach, stimulates appetite, gastric motility, growth hormone release, and regulates energy metabolism [97, 98]. Its upregulation may reflect an adaptive hormonal response aimed at boosting gastric function and feed intake efficiency in response to dietary fiber. Members of the sodium-hydrogen exchanger protein family, *SLC9A4* and *SLC9A3*, contribute to transepithelial sodium and water absorption, intracellular pH regulation, cellular volume regulation, and acid-base balance [99]. Sodium voltage-gated channel beta subunit 3 (*SCN3B*) modulates voltage-gated sodium channels, influencing membrane excitability [60]. The coordinated upregulation of these transporters in the pancreas of SB-fed broilers may indicate enhanced epithelial ion exchange capacity and acid-base balance, potentially reflecting adaptive responses to the increased fermentative and mechanical demands of an insoluble-fiber diet. These physiological responses underpin the higher nutrient and energy digestibility and utilization observed in SB-fed broilers by Kheravii et al. [16] and the superior performance in SB-fed broilers in this study.

Additionally, the term response to oxidative stress (GO:0006979) was enriched with *DUOX2*,* GPX2*, *EPX*, and *PTGS2*. Interestingly, three of these DEGs (*DUOX2*,* EPX*, and *PTGS2*), which were enriched in this biological process, were also enriched in two molecular function terms, heme binding (GO:0020037) and peroxidase activity (GO:0004601), likely acting as heme-dependent peroxidases. Heme is an iron-bound porphyrin complex that serves as a vital cofactor in various biological processes, including energy metabolism and detoxification [100]. Peroxidases, generally heme-containing enzymes, use H_2_O_2_ as a substrate to drive several biological processes, including the oxidation of ROS, xenobiotic detoxification, innate immunity, hormone biosynthesis, and inflammatory disease pathogenesis [101]. In SB-fed broilers, the upregulation of *DUOX2*, which produces H₂O₂ for redox signaling and mucosal innate immunity [87], and glutathione peroxidase 2 (*GPX2*), which converts H₂O₂ to water to maintain redox balance [102], likely reflects a proactive, homeostatic adaptation to mild oxidative cues from fiber fermentation. The upregulation of prostaglandin-endoperoxide synthase 2 (*PTGS2*, also known as *COX-2*), an enzyme involved in prostaglandin synthesis and inflammation [103, 104], indicates potential modulation of immune and metabolic signaling pathways in response to SB inclusion. Together, the coordinated induction of these terms, enriched with *DUOX2*, *GPX2*, and *PTGS2*, which are upregulated in the pancreas of SB-fed broilers, suggests improved mucosal defense, redox signaling, and metabolic support, without showing signs of oxidative stress.

Interestingly, membrane (GO:0016020), a cellular component, was the most significantly enriched term with the highest number of DEGs in both the jejunum and pancreas in this study. This term refers to a lipid bilayer, along with all the proteins and protein complexes embedded and attached to it. The function of a membrane protein is reflected by how it associates with the lipid bilayer [105]. This enrichment, involving genes that encode membrane-bound transporters, receptors, enzymes, and structural proteins, suggests a coordinated cellular adaptation to SB supplementation. The enrichment likely reflects tissue-wide and cellular efforts to regulate nutrient transport, ion exchange, immune signaling, and barrier integrity in response to the microbial fermentative activities induced by dietary fiber. For example, in the jejunum and pancreas, membrane-associated DEGs such as *TRPM3* (Ca²⁺ influx), *SLC16A4* (nutrient transport), *GAL3ST2* (sulfation of mucin glycans), *RBP4A* (retinol transport), and *FFAR4* (fatty acid sensing) indicate tissue-specific molecular adjustments to support epithelial maintenance and digestive efficiency of insoluble fiber as discussed above. These transcriptomic and physiological patterns align with previous findings [16] showing improved nutrient digestibility and utilization in SB-fed broilers and may represent molecular mechanisms contributing to those observed performance outcomes.

## Conclusion

This study demonstrates that SB supplementation induces beneficial transcriptomic responses in the jejunum and pancreas of broiler chickens, indicating coordinated molecular adaptations that may enhance immunity, nutrient utilization, and overall performance. The upregulation of genes related to mineral and energy transport (e.g., *TRPM3*, *SLC16A4*, *FFAR4*,* RBP4A*), epithelial integrity (*WNT9A*, *TFF3*, *AGR2*), redox and immune defense (*DUOX2*, *GPX2*, *MHCY6*), and growth regulation (*POU1F1*) indicates transcriptional changes that support gut health, nutrient transport, metabolism, and growth in SB-fed broilers. Additionally, several key biological processes that are significantly enriched may highlight the functional pathways involved in broilers’ physiological adaptation to SB supplementation. Overall, these findings provide insight into how dietary insoluble fiber may influence host physiology and offer a molecular basis for including alternative, fiber-rich feed ingredients, such as SB, in sustainable poultry production. Future studies should integrate omics-based evidence with broader assessments of physiological, metabolic, and performance traits and explore SB inclusion with other feed additives in low-protein diets to evaluate their impact on feed efficiency and sustainable broiler production.

## Supplementary Information

Supplementary Material 1. Supplementary Table 1: Differentially expressed genes in the jejunum of broilers fed the control diet compared to those fed the SB diet.

Supplementary Material 2. Supplementary Table 2: Differentially expressed genes in the pancreas of broilers fed the control diet compared to those fed the SB diet.

Supplementary Material 3. Supplementary Table 3: Gene ontology enrichment analysis of the DEGs in the pancreas.

## Data Availability

The raw and normalized count data for all samples have been deposited in the National Center for Biotechnology Information (NCBI) Gene Expression Omnibus (GEO) under accession number GSE330384 (https://www.ncbi.nlm.nih.gov/geo/query/acc.cgi?acc=GSE330384). Supplementary results, including differentially expressed genes and gene ontology enrichment analysis, are included in the Additional files.

## Abbreviations

SB: Sugarcane bagasse
SID: Standard ileal digestible
NSP: Non-starch polysaccharide
ME: Metabolizable energy
SEM: Standard error of mean
WG: Weight gain
FI: Feed intake
FCR: Feed conversion ratio
RNA-seq: RNA sequencing
DEGs: Differentially expressed genes
GO: Gene ontology
KEGG: Kyoto Encyclopedia of Genes and Genomes
SCFA: Short-chain fatty acids
RIN: RNA integrity number
PCA: Principal component analysis
DAVID: Database for Annotation, Visualization, and Integrated Discovery
RBP4A: Retinol-binding protein 4 A
*GAL3ST2*: Galactose-3-O-sulfotransferase 2
*WNT9A*: WNT family member 9 A
*SBSPON*: Somatomedin B and thrombospondin type 1 domain containing
*TFF3*: Trefoil factor family member 3
*AGR2*: Anterior gradient 2
*DUOX2*: Dual oxidase 2
*GPX2*: Glutathione peroxidase 2
*PTGS2*: Prostaglandin-endoperoxide synthase 2
*TRPM3*: Transient receptor potential melastatin-3
*SLC16A4*: Solute carrier family 16 member 4
*FFAR4*: Free fatty acid receptor 4
*MHCY6*: Major histocompatibility complex
*POU1F1*: Pituitary-specific positive transcription factor 1
