# Supplementary material for: Jejunal and pancreatic transcriptomic adaptations underpin enhanced performance in broilers fed sugarcane bagasse-supplemented diets
Source: BMC Genomics. 2026 May 22;27:620. doi: 10.1186/s12864-026-12978-3 (PMC13371210; doi:10.1186/s12864-026-12978-3)
Supplement: Supplementary file 1 — Supplementary Material 1. Supplementary Table 1: Differentially expressed genes in the jejunum of broilers fed the control diet compared to those fed the SB diet. [file 12864_2026_12978_MOESM1_ESM.docx]

**Supplementary Table 1. Differentially expressed genes in the jejunum of broilers fed the control diet compared to those fed the SB diet.**

| **Gene symbol** | **logFC^1^** | ***P*-value** | **Direction^2^** |
| --- | --- | --- | --- |
| *ENSGALG00010001529* | 1.744 | 0.001 | Upregulated |
| *SNPH* | 1.556 | 0.001 | Upregulated |
| *ENSGALG00010003520* | 1.412 | 0.003 | Upregulated |
| *FAM179A* | 1.401 | 0.005 | Upregulated |
| *LOC112530310* | 1.436 | 0.007 | Upregulated |
| *ENSGALG00010005303* | 1.583 | 0.009 | Upregulated |
| *ENSGALG00010016688* | 1.129 | 0.010 | Upregulated |
| *ENSGALG00010021837* | 1.048 | 0.011 | Upregulated |
| *KIF6* | 1.013 | 0.017 | Upregulated |
| *LOC112531163* | 1.174 | 0.019 | Upregulated |
| *LOC107050135* | 1.196 | 0.019 | Upregulated |
| *GLDN* | 1.621 | 0.020 | Upregulated |
| *KCND3* | 1.111 | 0.023 | Upregulated |
| *RASSF10* | 1.078 | 0.026 | Upregulated |
| *CA7* | 1.100 | 0.035 | Upregulated |
| *CHIR-B3* | 1.025 | 0.044 | Upregulated |
| *ENSGALG00010004383* | 1.167 | 0.045 | Upregulated |
| *ENSGALG00010012331* | 1.810 | 0.048 | Upregulated |
| *LOC121109863* | -1.423 | 0.002 | Downregulated |
| *TRPM3* | -1.594 | 0.003 | Downregulated |
| *LOC121106487* | -2.281 | 0.004 | Downregulated |
| *LOC415478* | -1.034 | 0.007 | Downregulated |
| *SBSPON* | -1.282 | 0.007 | Downregulated |
| *LOC121106936* | -2.277 | 0.008 | Downregulated |
| *SLC16A4* | -1.546 | 0.012 | Downregulated |
| *PITX3* | -1.256 | 0.014 | Downregulated |
| *WNT9A* | -3.440 | 0.022 | Downregulated |
| *ENSGALG00010011938* | -1.170 | 0.022 | Downregulated |
| *ENSGALG00010012827* | -1.391 | 0.022 | Downregulated |
| *ENSGALG00010018574* | -1.144 | 0.032 | Downregulated |
| *PRDM8* | -2.325 | 0.033 | Downregulated |
| *ENSGALG00010015567* | -1.171 | 0.034 | Downregulated |
| *VEPH1* | -1.903 | 0.036 | Downregulated |
| *ZNFY1* | -2.275 | 0.038 | Downregulated |
| *MHCY6* | -1.765 | 0.039 | Downregulated |
| *TTC24* | -1.230 | 0.039 | Downregulated |
| *AJAP1* | -1.800 | 0.040 | Downregulated |
| *CCR8L* | -1.195 | 0.042 | Downregulated |
| *POU1F1* | -1.470 | 0.044 | Downregulated |
| *KRT24* | -1.005 | 0.048 | Downregulated |
| *GAL3ST2* | -1.695 | 0.049 | Downregulated |

**^1^** logFC = log_2_ fold change

^2^ Direction: Indicates whether the gene is upregulated or downregulated. Upregulated genes are highly expressed in broilers fed the control diet than in SB-fed broilers, whereas downregulated genes are expressed at lower levels in control-fed broilers compared to those fed the SB diet
