# Supplementary material for: Jejunal and pancreatic transcriptomic adaptations underpin enhanced performance in broilers fed sugarcane bagasse-supplemented diets
Source: BMC Genomics. 2026 May 22;27:620. doi: 10.1186/s12864-026-12978-3 (PMC13371210; doi:10.1186/s12864-026-12978-3)
Supplement: Supplementary file 2 — Supplementary Material 2. Supplementary Table 2: Differentially expressed genes in the pancreas of broilers fed the control diet compared to those fed the SB diet. [file 12864_2026_12978_MOESM2_ESM.docx]

**Supplementary Table 2. Differentially expressed genes in the pancreas of broilers fed the control diet compared to those fed the SB diet.**

| **Gene symbol** | **logFC^1^** | ***P*-value** | **Direction^2^** |
| --- | --- | --- | --- |
| *LOC107057295* | 1.118 | 0.000 | Upregulated |
| *ZNF804B* | 1.213 | 0.001 | Upregulated |
| *SLC12A5* | 1.354 | 0.004 | Upregulated |
| *CFAP45* | 1.359 | 0.005 | Upregulated |
| *ENSGALG00010023711* | 4.816 | 0.007 | Upregulated |
| *RFLNA* | 1.611 | 0.007 | Upregulated |
| *ENSGALG00010000628* | 1.692 | 0.008 | Upregulated |
| *ENSGALG00010024066* | 1.924 | 0.008 | Upregulated |
| *LOC768817* | 4.967 | 0.009 | Upregulated |
| *DUSP6* | 1.108 | 0.009 | Upregulated |
| *ENSGALG00010000731* | 2.087 | 0.010 | Upregulated |
| *CGTL* | 1.329 | 0.011 | Upregulated |
| *PLEKHM3* | 1.164 | 0.012 | Upregulated |
| *EPX* | 1.080 | 0.013 | Upregulated |
| *MYH7* | 1.406 | 0.013 | Upregulated |
| *ENSGALG00010012676* | 6.478 | 0.013 | Upregulated |
| *ENSGALG00010017382* | 1.039 | 0.014 | Upregulated |
| *CFAP100* | 1.706 | 0.015 | Upregulated |
| *SYNDIG1L* | 1.442 | 0.016 | Upregulated |
| *ENSGALG00010000598* | 1.451 | 0.017 | Upregulated |
| *CARF* | 1.102 | 0.018 | Upregulated |
| *SPINK7* | 3.044 | 0.020 | Upregulated |
| *DNAJB8* | 3.027 | 0.021 | Upregulated |
| *C15orf39* | 1.368 | 0.021 | Upregulated |
| *IL12A* | 1.512 | 0.021 | Upregulated |
| *ENSGALG00010018624* | 4.812 | 0.023 | Upregulated |
| *GBP* | 1.479 | 0.030 | Upregulated |
| *EVA1CL* | 1.160 | 0.034 | Upregulated |
| *ENSGALG00010012836* | 3.103 | 0.034 | Upregulated |
| *ENSGALG00010007387* | 1.071 | 0.036 | Upregulated |
| *LOC112530140* | 1.058 | 0.037 | Upregulated |
| *IL13RA2* | 1.179 | 0.041 | Upregulated |
| *GDAP1* | 1.526 | 0.042 | Upregulated |
| *HOXD3* | 1.084 | 0.043 | Upregulated |
| *ENSGALG00010003264* | 1.767 | 0.043 | Upregulated |
| *AHR1B* | 1.622 | 0.044 | Upregulated |
| *ENSGALG00010023042* | -2.581 | 0.001 | Downregulated |
| *ENSGALG00010023878* | -3.318 | 0.001 | Downregulated |
| *WNT7A* | -2.376 | 0.001 | Downregulated |
| *CRISP2* | -3.276 | 0.001 | Downregulated |
| *ENSGALG00010001707* | -3.025 | 0.001 | Downregulated |
| *DUOX2* | -2.943 | 0.001 | Downregulated |
| *ENSGALG00010002378* | -1.446 | 0.001 | Downregulated |
| *SCN3B* | -1.081 | 0.002 | Downregulated |
| *TFF3* | -2.013 | 0.002 | Downregulated |
| *SPTSSB* | -2.304 | 0.003 | Downregulated |
| *LOC107052718* | -6.490 | 0.003 | Downregulated |
| *ENSGALG00010028520* | -2.396 | 0.003 | Downregulated |
| *AGR2* | -1.851 | 0.003 | Downregulated |
| *FFAR4* | -1.928 | 0.004 | Downregulated |
| *DUOXA1L* | -3.160 | 0.004 | Downregulated |
| *SLC28A3* | -2.270 | 0.004 | Downregulated |
| *GATD3AL2* | -2.567 | 0.004 | Downregulated |
| *ENSGALG00010007006* | -2.309 | 0.004 | Downregulated |
| *SCTR* | -1.617 | 0.005 | Downregulated |
| *SPP1* | -1.473 | 0.005 | Downregulated |
| *CES1L1* | -1.259 | 0.006 | Downregulated |
| *ENSGALG00010004778* | -2.096 | 0.006 | Downregulated |
| *LVRN* | -1.227 | 0.006 | Downregulated |
| *AvBD10* | -1.686 | 0.006 | Downregulated |
| *VNN2* | -1.521 | 0.007 | Downregulated |
| *ABCB5* | -1.206 | 0.008 | Downregulated |
| *RBP4A* | -1.856 | 0.009 | Downregulated |
| *ENSGALG00010007182* | -1.348 | 0.009 | Downregulated |
| *ENSGALG00010001560* | -2.664 | 0.009 | Downregulated |
| *PCP4* | -2.030 | 0.010 | Downregulated |
| *OTOF* | -3.109 | 0.010 | Downregulated |
| *POLN* | -1.305 | 0.010 | Downregulated |
| *ENSGALG00010001816* | -2.889 | 0.010 | Downregulated |
| *ENSGALG00010016898* | -3.101 | 0.011 | Downregulated |
| *PTGS2* | -1.306 | 0.011 | Downregulated |
| *FGFBP1* | -2.386 | 0.011 | Downregulated |
| *RYR2* | -1.835 | 0.012 | Downregulated |
| *CAPN8* | -1.524 | 0.012 | Downregulated |
| *SLC9A4* | -2.673 | 0.013 | Downregulated |
| *LOC121113331* | -1.539 | 0.014 | Downregulated |
| *ADAM28* | -1.281 | 0.014 | Downregulated |
| *SLC9A3* | -1.959 | 0.015 | Downregulated |
| *GHRL* | -1.142 | 0.015 | Downregulated |
| *GALR1* | -3.165 | 0.015 | Downregulated |
| *CAPN9* | -2.184 | 0.015 | Downregulated |
| *MMP7* | -2.498 | 0.015 | Downregulated |
| *LOC770705* | -1.681 | 0.016 | Downregulated |
| *ENSGALG00010011074* | -2.658 | 0.017 | Downregulated |
| *KRT40* | -2.688 | 0.017 | Downregulated |
| *ROS1* | -1.639 | 0.019 | Downregulated |
| *ARL14* | -1.356 | 0.019 | Downregulated |
| *BMX* | -1.541 | 0.019 | Downregulated |
| *MYO7B* | -1.736 | 0.020 | Downregulated |
| *ENSGALG00010007126* | -1.726 | 0.022 | Downregulated |
| *MHCY6* | -1.377 | 0.022 | Downregulated |
| *EHF* | -1.245 | 0.023 | Downregulated |
| *ENSGALG00010001726* | -1.314 | 0.023 | Downregulated |
| *LAPTM4B* | -1.168 | 0.023 | Downregulated |
| *ENSGALG00010015138* | -1.633 | 0.024 | Downregulated |
| *CHAT* | -1.246 | 0.025 | Downregulated |
| *RNLS* | -1.234 | 0.025 | Downregulated |
| *LOC107049808* | -1.725 | 0.026 | Downregulated |
| *SLC22A13L* | -1.612 | 0.028 | Downregulated |
| *ATP10B* | -1.760 | 0.028 | Downregulated |
| *TTN* | -1.782 | 0.029 | Downregulated |
| *DPP6* | -1.250 | 0.029 | Downregulated |
| *COL17A1* | -1.122 | 0.030 | Downregulated |
| *SOSTDC1* | -1.167 | 0.030 | Downregulated |
| *LOC100857292* | -2.027 | 0.030 | Downregulated |
| *HPX* | -1.140 | 0.030 | Downregulated |
| *RBP* | -1.015 | 0.034 | Downregulated |
| *MCOLN2* | -1.015 | 0.034 | Downregulated |
| *ENSGALG00010024706* | -1.897 | 0.037 | Downregulated |
| *ENSGALG00010007123* | -1.683 | 0.038 | Downregulated |
| *C4orf54* | -1.941 | 0.038 | Downregulated |
| *LOC121106936* | -1.193 | 0.039 | Downregulated |
| *ENSGALG00010002366* | -1.307 | 0.039 | Downregulated |
| *BCHE* | -1.119 | 0.039 | Downregulated |
| *CDH23* | -1.277 | 0.040 | Downregulated |
| *CKMT1B* | -1.054 | 0.042 | Downregulated |
| *GPX2* | -1.249 | 0.042 | Downregulated |
| *ENTPD8L2* | -1.424 | 0.042 | Downregulated |
| *CLRN3* | -1.347 | 0.043 | Downregulated |
| *ENSGALG00010003368* | -1.059 | 0.043 | Downregulated |
| *PCDH8* | -1.647 | 0.044 | Downregulated |
| *OVST* | -1.430 | 0.045 | Downregulated |
| *MYOM2* | -1.461 | 0.045 | Downregulated |
| *KCNH6* | -1.089 | 0.047 | Downregulated |
| *CYP2C23a* | -1.413 | 0.050 | Downregulated |

**^1^** logFC = log_2_ fold change

^2^ Direction: Indicates whether the gene is upregulated or downregulated. Upregulated genes are highly expressed in broilers fed the control diet than in SB-fed broilers, while downregulated genes are expressed at lower levels in control-fed broilers compared to those fed the SB diet
