# Supplementary material for: Jejunal and pancreatic transcriptomic adaptations underpin enhanced performance in broilers fed sugarcane bagasse-supplemented diets
Source: BMC Genomics. 2026 May 22;27:620. doi: 10.1186/s12864-026-12978-3 (PMC13371210; doi:10.1186/s12864-026-12978-3)
Supplement: Supplementary file 3 — Supplementary Material 3. Supplementary Table 3: Gene ontology enrichment analysis of the DEGs in the pancreas. [file 12864_2026_12978_MOESM3_ESM.docx]

**Supplementary Table 3. Gene ontology enrichment analysis of the DEGs in the pancreas**

| Category | Term | Count | *P*-value | Genes |
| --- | --- | --- | --- | --- |
| Biological Process | GO:0015074~DNA integration | 5 | 0.001 | *LOC107052718, LOC770705, ENSGALG00010007387, ENSGALG00010003264, ENSGALG00010004778* |
|  | GO:0016032~viral process | 4 | 0.006 | *ENSGALG00010003264, ENSGALG00010023711, ENSGALG00010018624, ENSGALG00010012836* |
|  | GO:0006508~proteolysis | 9 | 0.007 | *ADAM28, CAPN9, DPP6, MMP7, LVRN, CAPN8, LOC768817, ENSGALG00010012676, ENSGALG00010003264* |
|  | GO:0043627~response to extrogen | 3 | 0.009 | *GHRL, RBP4A, WNT7A* |
|  | GO:0006979~response to oxidative stress | 4 | 0.010 | *EPX, PTGS2, DUOX2, GPX2* |
|  | GO:0019373~epoxygenase P450 pathway | 2 | 0.013 | *CYP2C23a* |
|  | GO:0006814~sodium ion transport | 3 | 0.028 | *SLC9A4, SLC9A3, SCN3B* |
|  | GO:0006278~RNA-templated DNA biosynthetic process | 2 | 0.031 | *LOC107052718, LOC770705* |
|  | GO:0006805~xenobiotic metabolic process | 3 | 0.036 | *CYP2C23a, AHR1B* |
|  | GO:0001696~gastric acid secretion | 2 | 0.043 | *GHRL, SLC9A4* |
|  | GO:0030277~maintenance of gastrointestinal epithelium | 2 | 0.043 | *RBP4A, TFF3* |
|  | GO:0032691~negative regulation of interleukin-1 beta production | 2 | 0.061 | *GHRL, FFAR4* |
|  | GO:0086005~ventricular cardiac muscle cell action potential | 2 | 0.067 | *SCN3B, RYR2* |
|  | GO:0010460~positive regulation of heart rate | 2 | 0.073 | *SCN3B, RYR2* |
|  | GO:0090336~positive regulation of brown fat cell differentiation | 2 | 0.073 | *PTGS2, FFAR4* |
|  | GO:0006082~organic acid metabolic process | 2 | 0.096 | *CYP2C23a* |
|  | GO:0006885~regulation of PH | 2 | 0.096 | *SLC9A4, SLC9A3* |
| Cellular Component | GO:0005615~extracellular space | 19 | 0.000 | *GHRL, RBP4A, WNT7A, EPX, TFF3, COL17A1, BCHE, HPX, SPINK7, SPP1, AGR2, SOSTDC1, MHCY6, IL12A, OVST, CRISP2, AvBD10, LOC768817, ENSGALG00010023042,* |
|  | GO:0005576~extracellular region | 18 | 0.000 | *GHRL, WNT7A, COL17A1, BCHE, HPX, SPP1, IL12A, OVST, FGFBP1, CRISP2, AvBD10, RNLS, CFAP45, LOC768817, ENSGALG00010001707, ENSGALG00010023878, ENSGALG00010023042, ENSGALG00010024706* |
|  | GO:0016020~membrane | 34 | 0.035 | *DPP6, RBP4A, WNT7A, SLC9A4, FFAR4, COL17A1, BCHE, EVA1CL, SYNDIG1L, GALR1, SCTR, ATP10B, SLC12A5, ENTPD8L2, ABCB5, SLC28A3, CGTL, KCNH6, OTOF, DUOXA1L, MYO7B, CLRN3, SLC22A13L, IL13RA2, ROS1, LOC107057295, LOC121106936, ENSGALG00010011074, ENSGALG00010001560, ENSGALG00010012836, ENSGALG00010001726, ENSGALG00010001707, ENSGALG00010000628, ENSGALG00010001816* |
| Molecular Function | GO:0003964~RNA-directed DNA polymerase activity | 6 | 0.000 | *LOC107052718, LOC770705, ENSGALG00010003264, ENSGALG00010004778, ENSGALG00010012676, ENSGALG00010007387* |
|  | GO:0004523~RNA-DNA hybrid ribonuclease activity | 5 | 0.003 | *LOC107052718, LOC770705, ENSGALG00010003264, ENSGALG00010004778, ENSGALG00010007387* |
|  | GO:0020037~heme binding | 5 | 0.009 | *EPX, PTGS2, DUOX2, CYP2C23a* |
|  | GO:0004601~peroxidase activity | 3 | 0.011 | *EPX, PTGS2, DUOX2* |
|  | GO:0030246~carbohydrate binding | 5 | 0.015 | *SPINK7, EVA1CL, LOC121113331, ENSGALG00010007123, ENSGALG00010007182* |
|  | GO:0008270~zinc ion binding | 11 | 0.016 | *MMP7, LVRN, LOC107052718, LOC770705, ENSGALG00010003264, ENSGALG00010004778, ENSGALG00010023711, ENSGALG00010018624, ENSGALG00010012836, ENSGALG00010012676, ENSGALG00010007387* |
|  | GO:0008392~arachidonate epoxygenase activity | 2 | 0.019 | *CYP2C23a* |
|  | GO:0015385~sodium: proton antiporter activity | 2 | 0.069 | *SLC9A4, SLC9A3* |
|  | GO:0004867~serine-type endopeptidase inhibitor activity | 3 | 0.083 | *SPINK7, OVST, ENSGALG00010023042* |
